# Treatment of visual axis opacification and secondary membranes with Nd:YAG laser after pediatric cataract surgery under intranasal sedation

**DOI:** 10.3389/fped.2023.1124030

**Published:** 2023-04-12

**Authors:** Pingjun Chang, Siyan Li, Dandan Wang, Chaoqiao Chen, Yana Fu, Man Hu, Shuyi Qian, Yun-e Zhao

**Affiliations:** ^1^ National Clinical Research Center for Ocular Diseases, Eye Hospital, Wenzhou Medical University, Wenzhou, China; ^2^ Eye Hospital of Wenzhou Medical University Hangzhou Branch, Hangzhou, China

**Keywords:** Nd:YAG laser, pediatric cataract, visual axis opacification, secondary membrane, intranasal 2 dexmedetomidine sedation

## Abstract

**Purpose:**

To describe neodymium-doped yttrium-aluminum-garnet (Nd:YAG) laser treatment of visual axis opacification and secondary membranes in pediatric patients with cataracts under intranasal dexmedetomidine sedation.

**Methods:**

Twenty eyes of 17 patients with secondary membrane formation after cataract extraction were enrolled in this study. Intranasal dexmedetomidine sedation (3 ug/kg) was administered, and Nd:YAG laser (Ellex Super Q, Adelaide, Australia) procedures were performed with children in the sitting position with their chin supported on a laser delivery slit lamp. Preoperative and postoperative visual acuities were documented, and medical records were reviewed.

**Results:**

The age of the patients ranged from 5 to 83 months (31.82 ± 27.73). Nineteen (95.0%) eyes had congenital cataracts and one (5.0%) had a traumatic cataract. Nd:YAG laser treatment of VAO with ten (50.0%) eyes, pupillary membranes with three (15.0%) eyes, pupillary cortical proliferation with six (30.0%) eyes, and anterior capsule contraction with one (5.0%) eye. Five (25.0%) eyes demonstrated visual acuity improvement, whereas six (30.0%) eyes remained unchanged after laser treatment. The recurrence rate was 30.0% and four eyes underwent a second Nd:YAG membranectomy. No side effects or tolerances due to sedative drugs were observed.

**Conclusion:**

Nd:YAG laser membranectomy under intranasal dexmedetomidine sedation was safely performed in children as young as 5 months old in a sitting position. This approach facilitates patient convenience, doctor proficiency, and cost reductions. Patients with recurrence can be treated by repeating the procedure.

## Introduction

The development of contemporary microscopic equipment and surgical technology has improved the success and safety of pediatric cataract surgery. Postoperative complications can occur even with an adequate operative procedure. Increased vascular permeability in pediatric patients can easily cause fibrinoid inflammation, resulting in the formation of fibrous membranes (1). Secondary membranes occur when the inflammatory fibrous membranes are not absorbed thoroughly in the early postoperative period, either in the aphakic eyes or in the intraocular lens (IOL) eyes. Secondary membranes can present along the posterior surface of the iris, capsule remnants, and anterior vitreous face (2). Severe inflammatory reactions and excessive fibrosis of the anterior capsule can reduce the size of the anterior capsule opening, causing decentration and tilt of the IOL. Due to the drastic proliferation of lens epithelial cells, the clouding of the visual axis area formed in the middle and late postoperative stages. Visual axis opacification (VAO) leads to visual deprivation and results in irreversible amblyopia (3). Thus, secondary membranes and VAO add to the difficulty of achieving a good outcome.

Many surgical techniques have been used to reduce VAO and secondary membrane formation in pediatric cataract management. Posterior continuous curvilinear capsulorhexis (PCCC) remains the gold standard to prevent posterior capsular opacification (PCO) (4). Anterior vitrectomy plays a critical role in reducing VAO incidence (5). Despite many of these techniques, VAO and secondary membranes occur frequently. In particular, the younger the child is, the more likely VAO and secondary membranes will occur. VAO is the most common postoperative complication, and statistics have revealed the most common need for additional intraocular surgery was cleaning the VAO (6, 7). Therefore, early recognition and prompt treatment of postoperative complications are critical in visual development. Although some retrospective studies have reported the prevalence of secondary membrane formation after pediatric cataract surgery (2, 3, 8), only a few have focused on secondary membrane interventions. Neodymium-doped yttrium-aluminum-garnet (Nd:YAG) laser capsulotomy is the standard treatment for adults with PCO and was first described in 1985 to treat secondary membranes in the pediatric population (9). However, this slit-lamp delivery system can only be used in older cooperative children who receive treatment while seated. Atkinson et al. reported the management of secondary posterior capsular membranes in younger children under general anesthesia using a rotation laser delivery system (10). Nevertheless, Nd:YAG laser capsulotomy under general anesthesia has some disadvantages, including anesthesia risk, technical difficulties, unavailability of systems, and high cost.

In this study, we demonstrate the feasibility of performing Nd:YAG laser treatment in children of all ages in a sitting position with intranasal dexmedetomidine sedation. The current method applies to many types of postoperative complications including VAO, pupillary membranes, pupillary cortical proliferation, and anterior capsule contraction.

## Materials and methods

### Patients and study design

This was a consecutive, observational case series. Twenty eyes of 17 patients with cataracts were included in this study. The patients were divided into two groups: the “aphakic group” and the “pseudophakic group” (Table 1). All laser treatments were performed by two surgeons at the Eye Hospital of Wenzhou Medical University, Hangzhou, China, between June 2019 and March 2021. This study was approved by the Ethics Committee of the Eye Hospital of Wenzhou Medical University (ChiCTR1800014417). All patients underwent slit-lamp examination, and Teller visual acuity test or best-corrected visual acuity test.

**Table 1:** Demographic and clinical data of enrolled patients.

No.	Age (months)	Gender	Affected eye	Cataract type	Additional diagnoses	IOL implantation	IOL type	Target of Laser	Interval Between Laser and Final Surgery (Months)	Visual Acuity		IOP (mmHg)	
										Preoperation	Postoperation	Preoperation	One-week Postoperation
1	23	M	Right	Congenital	Amblyopia, Exotropia	None	/	Papillary cortical proliferation	17	20/1600	20/1600	14	17.5
2	9	M	Bilateral	Congenital	Amblyopia	None	/	Papillary cortical proliferation	7	20/1200	20/800	9.3/10	8.8/9.6
3	27	F	Right	Congenital	Amblyopia, PFV	None	/	Posterior VAO	9	N/A	N/A	13.5	15.1
4	15	M	Left	Congenital	Amblyopia	None	/	Posterior VAO	13	20/1200	20/1200	10.2/11.7	14.4/13.5
5	7	F	Left	Congenital	Amblyopia, Posterior capsule defect	None	/	Papillary cortical proliferation	6	N/A	N/A	9.7	8.7
6	65	M	Left	Congenital	Amblyopia, Nystagmus	Capsular bag	AcrySof SN60AT +30.0D	Posterior VAO	41	20/160	20/63	19.8	15.8
7	48	M	Right	Congenital	Iridocyclitis	Capsular bag	Proming A1-UV +30.0D	Posterior VAO	26	N/A	N/A	13.1	13.2
8	7	F	Left	Congenital	Amblyopia, Esotropia, Posterior capsule defect, PFV	None	/	Posterior VAO	4.5	20/1200	20/800	12.4	15.6
9	11	M	Right	Congenital	Amblyopia, Esotropia, Nystagmus, Anterior capsule defect, Posterior capsule defect	None	/	Posterior VAO	1.5	20/800	20/800	9.2	9.4
10	46	M	Bilateral	Congenital	Amblyopia, Anterior lenticonus, PFV	Ciliary sulcus	AMO AR40e + 16.0D/+16.0D	Secondary membrane	18	20/800	20/800	18.6/16.5	10.1/8.8
11	83	M	Right	Congenital	Amblyopia	Posterior capsule clamping	AMO AR40e + 24.0D	Posterior VAO	16	20/1200	20/1200	12	14.4
12	77	M	Bilateral	Congenital	Amblyopia, Esotropia, Nystagmus	Capsular bag	Proming A1-UV +19.0D/+19.0D	Posterior VAO	19	N/A	N/A	14.9/11.4	9.5/11.8
13	74	F	Left	Congenital	Amblyopia, Exotropia	Capsular bag	AMO AR40e + 11.0D/+14.5D	Posterior VAO	14	20/50	20/50	15.7	12
14	14	M	Left	Congenital	Amblyopia	None	/	Posterior VAO	10	N/A	20/800	10.3	16.4
15	23	M	Right	Traumatic	Amblyopia, Corneal perforating injury, Anterior iris synechia, Hyphema, Vitreous hemorrhage	Capsular bag	Proming A1-UV +25.0D	Secondary membrane	5	N/A	N/A	12.1	15.6
16	5	M	Bilateral	Congenital	Amblyopia	None	/	Papillary cortical proliferation	3	N/A	N/A	7.4	7.5
17	7	F	Right	Congenital	Amblyopia	None	/	Secondary membrane	1	N/A	N/A	9.3	13.3

### Preparation and operation

**Figure 1:**
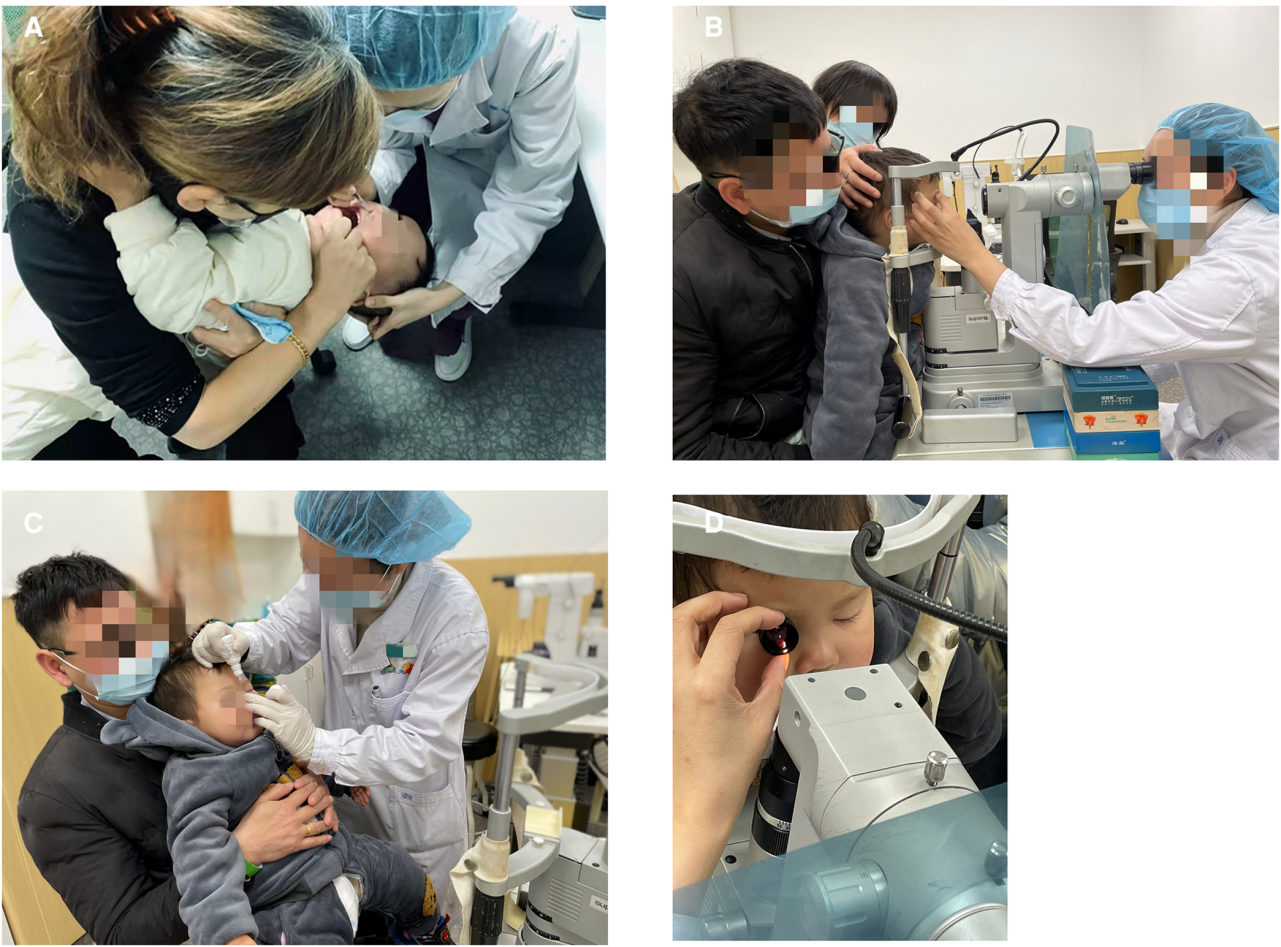
The procedure of Nd:YAG treatment under intranasal dexmedetomidine sedation. (**A**) Intranasal dexmedetomidine (3 ug/kg) sedation was administrated to the patient. (**B**) Topical anesthesia was applied to the patient. (**C,D**) The Nd:YAG laser procedure was performed with children in the sitting position with their chins supported on the laser delivery slit lamp.

Informed consent was obtained after the treatment procedure was explained to the parents in detail. Preoperative pupillary dilatation was performed using a combination of tropicamide 1.0% and phenylephrine hydrochloride 2.5%. Nd:YAG membranectomy was performed in the outpatient laser treatment room with children under intranasal dexmedetomidine sedation (Figure 1A). Vital signs, including pulse oximetry, blood pressure, oxygen saturation, and heart rate, were continuously monitored and recorded every ten minutes. The procedure for intranasal dexmedetomidine sedation was described in a previous article published by our institute (11). Dexmedetomidine (Ai Bei Ning; Jiang Su Heng Rui Medicine Co. Ltd, Jiangsu Province, China) was prepared at the concentration of 100 µg/ml and administered in 1 ml tuberculin syringe by the same anesthesiologist. The children were sedated with 3 ug/kg intranasal dexmedetomidine. After intranasal dexmedetomidine sedation was completed, topical anesthesia (proparacaine hydrochloride 0.5%) was conducted for every child to assure comfort (Figure 1B).

**Figure 2:**
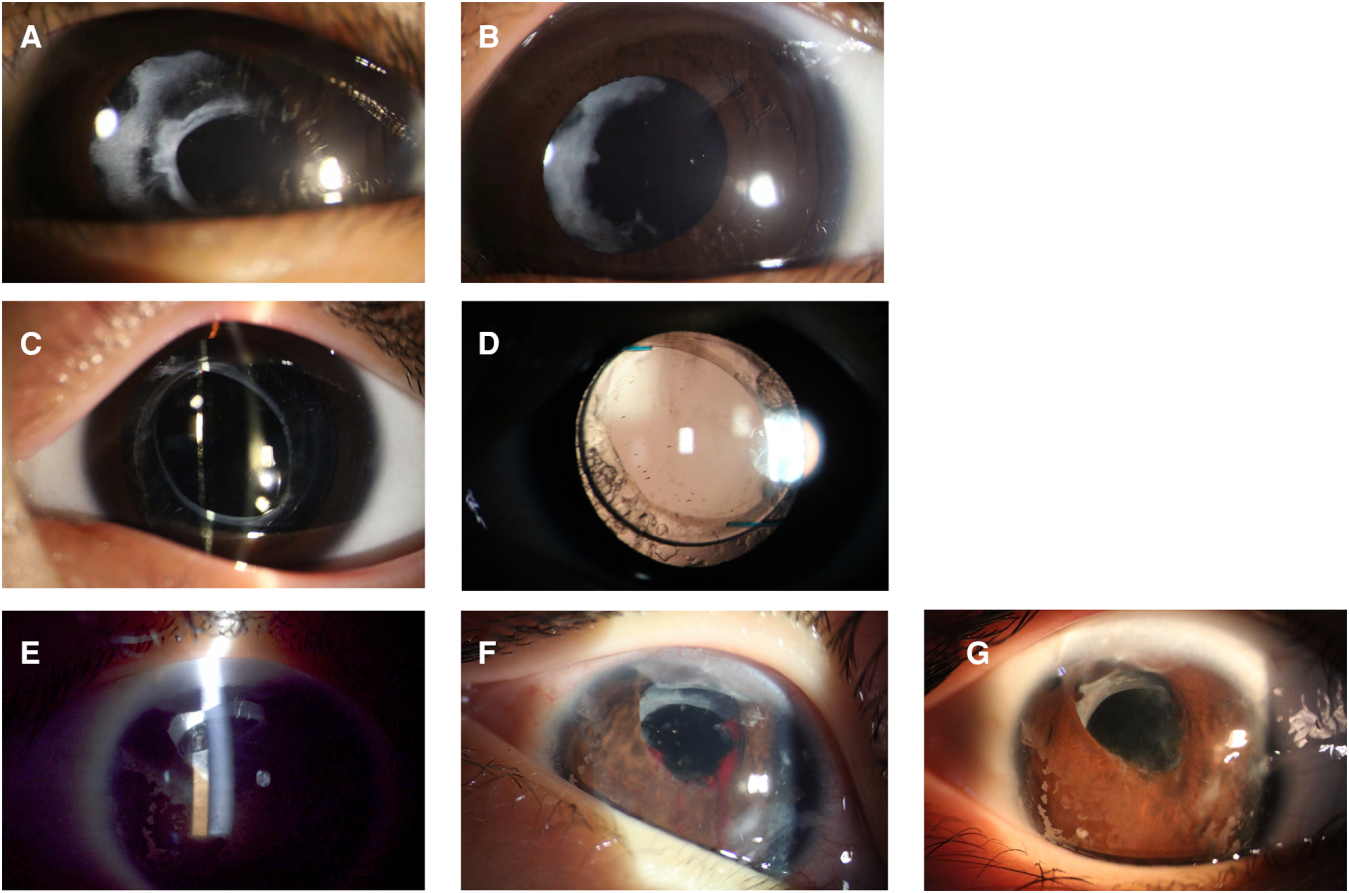
The preoperative (**A,C,E**) and postoperative (**B,D,G**) anterior segment configuration of patients. (**A,B**) Nd: YAG laser treatment of pupillary membranes along with anterior capsule remnants. (**C,D**) Nd:YAG laser posterior membranectomy was performed in a patient with posterior capsule capturing IOL implantation. (**E,F,G**) Nd:YAG laser treatment of anterior capsule contraction was performed in a patient with intracapsular IOL implantation, and iris hemorrhage occurred during treatment.

The Nd:YAG laser (Ellex Super Q, Adelaide, Australia) was used with children sitting with their chins supported on a laser delivery slit lamp (Figures 1C,D). Every child was accompanied by parents to assist with their position or to decrease stress. The use of contact lenses depends on the doctor's operating customs. The pattern of laser application depends on the nature and configuration of the membrane. When the target of laser membranectomy was the VAO, single bursts were applied to start at 0.8 mJ and then gradually increased until it was satisfied to create at least a 3-mm axial membranectomy. When the target was the pupillary membranes or the pupillary cortical proliferation, the Nd:YAG laser was applied to start at 1.0 mJ to target the densest areas of membrane formation and to avoid damage to the IOL. The energy level increased depending on the density of the membrane and proliferation. Anterior capsule contraction release was performed beginning at the margin of the anterior capsule and extending peripherally to the edge of the IOL optics. The energy levels were 1.5–2.0 mJ and 4–5 relaxing incisions were made. The preoperative and postoperative anterior segment configuration was recorded using a slit-lamp digital camera (Figure 2).

Patients received topical loteprednol etabonate 0.5% suspension four times daily for 7 days postoperatively. Initial follow-up examinations including slit-lamp examination, and Teller visual acuity test or best-corrected visual acuity test, intraocular pressure (IOP) was measured using a rebound tonometer (iCare, Vantaa, Finland) were performed within 7 days of the procedure. The follow-up duration ranged from 7 to 18 months. The medical records of 17 patients were reviewed.

### Statistical analysis

Data are presented as mean ± standard deviation and range, indicating the minimum and maximum values. Differences between the two groups were analyzed using a two-tailed Student's t-test. The variables were evaluated for bivariate relationships with other variables by examining Pearson's correlation coefficient for statistical significance. The criterion for statistical significance was set at *P* < 0.05.

## Results

### The observations of the visual axis opacification and secondary membrane formation in the eyes

**Figure 3:**
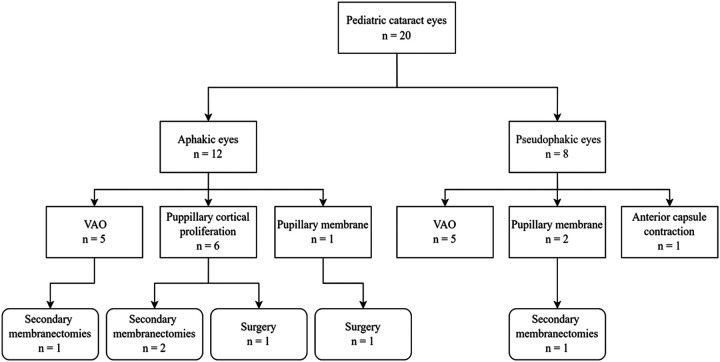
Targets of Nd:YAG laser treatment for the enrolled patients.

Twelve boys and 5 girls, ranging in age from 5 months to 83 months (31.82 ± 27.73) were enrolled in the current study. In the 20 eyes of 17 children, 19 (95.0%) eyes had congenital cataracts and one (5.0%) had a traumatic cataract. Nd:YAG laser treatment of VAO has performed with ten (50.0%) eyes, pupillary membranes with three (15.0%) eyes, pupillary cortical proliferation with six (30.0%) eyes, and anterior capsule contraction with one (5.0%) eye (Figure 3). The laser was applied to the right eye in eight children, to the left eye in six children, and bilateral eyes in three children.

Five (25.0%) eyes demonstrated VAO and 2 (10.0%) pupillary membranes after cataract extraction with IOL implantation. Five aphakic (25.0%) eyes showed VAO and six (30.0%) cortical proliferation. The lensectomy and posterior capsulotomy combined with limited anterior vitrectomy was performed in 17 (85.0%) eyes, five of which received stage II IOL implantation. The cataract phacoaspiration and posterior capsulorhexis and limited anterior vitrectomy combined with IOL implantation were performed in 3 (15.0%) eyes. The mean interval between laser and final surgery in eyes with and without IOL implantation was 19.86 ± 11.25 (5–41) and 7.20 ± 5.14 (1–17) months respectively. There was no linear relationship between surgical age and laser intervals.

Visual acuities were recorded in 11 eyes and could not be assessed in seven uncooperative children. Five (25.0%) eyes demonstrated improvement, whereas six (30.0%) eyes remained unchanged after laser treatment. In these six eyes, visual impairment was related to amblyopia, and one eye was accompanied by nystagmus. There was a strong positive linear relationship between preoperative and postoperative visual acuity (*r* = 0.908, *p* < 0.01). There was no linear relationship between age at laser treatment and visual acuity improvement.

### Nd:YAG membranectomy prognoses and postoperative complications

Four (20%) eyes received more than twice Nd:YAG laser membranectomy, three of which were pseudophakic. The duration between laser treatments was 4.25 ± 3.95 (1–10) months. The recurrent membranes were treated using a second Nd:YAG membranectomy to clear the visual axis. Patient 10 developed iris hemorrhage during laser treatment, and as a result, relapses soon occurred (Figure 2F). Patient 12 and 17 required surgeries to remove the posterior membrane after laser treatment due to intense inflammatory exudation. The presence of intraocular hypertension, corneal edema, hyphema, IOL dislocation, secondary uveitis, and pupillary block glaucoma was not observed in this study.

## Discussion

In the current study, we report a non-general anesthesia Nd:YAG laser treatment for postoperative membranes in cataract children aged 5 months to 83 months. Management of VAO and secondary membranes affects outcomes of pediatric cataract surgery. Nd:YAG laser capsulotomy is the standard treatment for PCO in adults. This procedure has the benefits of efficiency, painlessness, and portability and is generally available to ophthalmologists. When this method is used to treat postoperative cataracts in children with VAO, it is limited by the fact that the children are too young to cooperate and must rely on general anesthesia.

In 1994, Atkinson et al. introduced a 90° rotatable Nd:YAG laser system (Microruptor III, Meridian AG) used to treat children under general anesthesia in the supine position (10). This laser equipment is not widely used, and the operation requires specialized training. Therefore, many scholars have attempted to use standard laser therapy based on general anesthesia at different positions. Longmuir et al. reported the positioning of patients after induction of anesthesia using an electric chair in the sitting position (12). A previous large-scale study summarized 87 cases of Nd:YAG laser capsulotomy in the operating room (13). This operation required patients to be in the supine position when general anesthesia and intubation were performed, and then in the lateral decubitus position for laser treatment. However, positioning patients under general anesthesia is probably a challenging task since the patient's head, neck, and the tracheal tube should be secured safely. It requires a scrub nurse, circulating nurse, and other additional staff members to assist in positioning the patient. Additionally, positioning patients under general anesthesia is associated with a variety of complications including hypotension and heart rate decline (14, 15). Many children need to undergo repeated laser capsulotomies due to the intense postoperative ocular inflammatory exudate, however, repeated general anesthesia involves risks associated with operational inconvenience and complications.

In the present study, we provided access to pediatric cataract patients and allowed the safe use of a standard Nd:YAG laser while the patient remained in a sitting position. Intranasal dexmedetomidine (3 ug/kg) sedation is a feasible and safe method of Nd:YAG laser treatment in outpatient practice. Dexmedetomidine is a central α2-adrenergic receptor agonist with analgesic and anxiolytic properties. Administration of dexmedetomidine by the intranasal route has become a popular technique for sedation in children currently. It may cause a reduction in heart rate and blood pressure, but there is little evidence that it causes respiratory depression (16). A previous study by our institute (11) suggested that the heart rate and blood oxygen saturation would decline after 30 min of intranasal sedation. Thus, vital signs, including pulse oximetry, blood pressure, oxygen saturation, and heart rate, were continuously monitored and recorded every 10 min after sedation was administered, and no other systemic complications were observed. The duration of the laser procedure was less than 20 min in our practice. We recommended that parents wipe their children's faces with a chilled towel 30 min after completion of the laser treatment and examination. Additionally, one of the nurses at our clinical center was dedicated to following up with the patient's parents by phone 48 h postoperatively to provide necessary assistance.

The recurrence of the secondary membrane was 25%–60% demonstrated in previous studies, and the majority of the patients with recurrence were treated with Nd:YAG laser two or three times (9, 17, 18). In our study, the recurrence rate was 30.0% (six eyes). Four eyes underwent a second Nd:YAG membranectomy under the same sedation approach to clear their visual axis. The other two children who had recurrent VAO that were too dense to operate laser membranectomy underwent surgeries. We noticed that iris hemorrhage during laser manipulation exacerbated the inflammatory exudate and led to a rapid recurrence of VAO. Drug resistance was not observed with repeated use of intranasal dexmedetomidine. Using this approach, patients who need repeated laser treatments do not need to be at risk of complications from general anesthesia multiple times, and the cost is much less. In our experience, the cost of treatment with intranasal dexmedetomidine is 80% less than that under general anesthesia. This breaks the barrier of the high costs of Nd:YAG laser treatment of postoperative secondary membranes in pediatric cataracts. Additionally, the positioning of patients can be easily monitored by their parents. The sitting position is more customary for doctors to operate the laser, as it is similar to the operation in adult patients. So that the laser can be focused in place to smoothly remove the membrane without damaging other eye structures or the IOLs. Therefore, Nd: YAG laser treatment can be used in pediatric hospitals or pediatric ophthalmology offices.

Maintaining a clear visual axis is key to the success of pediatric cataract surgery, and many surgical techniques have been proposed to delay the formation of secondary membranes (5). Previous studies indicated that VAO occurs in 30%–40% of cases of pediatric cataracts even after a primary posterior capsulorrhexis, with the rate being higher in younger age groups (19–21). In our clinical center, for children younger than 3 years old, the lensectomy and posterior capsulotomy combined with limited anterior vitrectomy was performed; for age between 3 and 8 years received cataract phacoaspiration and posterior capsulorhexis and limited anterior vitrectomy; and for the children older than 8 years who could cooperate to receive Nd: YAG laser capsulotomy, they just received cataract phacoaspiration and the posterior capsule and vitreous were left intact. The lensectomy, capsulotomy, and vitrectomy were all operated with the 23-gauge (23 G, 0.6 mm) vitrector through 2 corneal incisions (10 o'clock and 2 o'clock). However, because of the rapid proliferation and migration of lens epithelial cells across the central visual axis, VAO was still the first cause of postoperative complications. In the present study, the mean interval between laser and final surgery in eyes with and without IOL implantation was 19.86 ± 11.25 (5–41) and 7.20 ± 5.14 (1–17) months, respectively. Since there is a critical period of visual development, once this period is missed, it will be difficult to develop visual acuity improvement. Using this laser technique is possible to bring forward the time of laser treatment, giving children more favorable timing guarantees for visual stimulus and visual development. Based on the consideration that fighting inflammation is the key to preventing recurrence of VAO, we prescribed topical loteprednol etabonate 0.5% suspension four times daily for 7 days to the patients postoperatively. We did not measure IOP in the immediate post-operative period, considering that Nd: YAG laser capsulotomy does not cause IOP elevation in most patients. However, our center takes IOP measurements very seriously in the follow-up of each patient, and we measure IOP at one-week and one-month postoperative checkups and keep individual records for each patient.

In conclusion, our experience indicates that Nd:YAG laser membranectomy under intranasal dexmedetomidine sedation may be safely performed in children as young as 5 months old, in a sitting position. This procedure facilitates patient convenience, doctor proficiency, and cost reduction. Patients with recurrence can be treated by repeating the procedure. To the best of our knowledge, this is the first report of intranasal dexmedetomidine administration in a pediatric laser treatment practice. Although we did not observe side effects due to sedative drugs in this study, which was limited by the patient scale and duration, the indications for this approach should be further explored.

## Data Availability

The original contributions presented in the study are included in the article, further inquiries can be directed to the corresponding author.
